# Forces Which Make for Progress

**Published:** 1893-01

**Authors:** 


### FORCES WHICH MAKE FOR PROGRESS.

The growth of civilization and the elevation of peoples in a moral and intellectual sense is an ever-instructive problem. Nations have moved in the circle of development from savageism to a higher cultivation, and returned again to nearly the first estate, apparently but little better for the civilizing gymnastics. The circle has been the favorite figure of many of the philosophers of the ages. It presents many discouraging features to the optimist who is unwilling to see anything in the future but an unending advancement to higher and still higher perfections. The scientific thought very naturally drifts into this conception, as any loss from a point gained seems an impossible idea, and yet the world cannot fail to remember its "lost arts" and past periods of active scientific development.

The forces that underlie progress are subtle in their character, but within certain limits are irresistible. The gentle zephyr becomes the cyclone. The slight shock of the Leyden jar indicates a dynamic force to influence a universe.

The lines of force in the physical run parallel with the moral and intellectual, while the circles are ever changing and the orbit of thought presenting new phases, the impetus given being ever onward to a more orderly sequence and larger development.

The contemplation of the last decade of the century, thought-engendered by the new year, carries us back, very naturally, to the forces which have developed dentistry through its ten periods, each containing experiences of vital interest to the profession.

The dentistry of 1800 to 1810 was remarkable only for the fact that it exerted no perceptible influence for good or ill. The places where men labored as dental surgeons were practically shops, but they were building better than they knew, for the energy which was to travel through the century was even then silently at work.

Moving along decade by decade, the power widens and the environments improve, one period witnessing one advance and another still greater growth, one a period of college development, another of law, one great professional ability, and another enlarged theoretical acquirement. The force of intellectual progress has steadily advanced, few perceiving it, and to some it may be unintelligible, but it is ever making for a larger growth in the direction of a fuller professional life.

The world needs to be oftentimes reminded that the forces that make for progress are not impelled in one direction, but radiate everywhere, seemingly at times in diverse and antagonistic lines, yet the manifestations are ever tending to increasing mental power and professional strength. It is not alone in the classic shades that the world looks for its masters, but rather in the great active world, where frictional energy is being constantly developed, evolving new ideas, new relations, new activities.

He is most wise who can read the indications of accumulated power. Sooner or later this increase will result in the bursting of bonds, and revolutions will arise in the political, moral, religious, and even in the scientific world. When Wendell Phillips said, "Revolutions are not made, they come," he spoke a great truth. They are the accumulated force of long periods of apparent inertia.

The conservative, or not deep-thinking element in dentistry must certainly see that the close of the last decade of the century means the centralizing of all these periods of active thought, and the breaking away from the old, and with this change revolution of ideas will come. Selfish indulgence in ease, commercial alliances, want of professional enthusiasm, all must give place to a higher conception of duty than now exists.

The future of dentistry is yet an unsolved problem, but in whatever direction it may be led it will advance only in the broadest and best sense, by a close adherence to those laws which have developed individuals and nations, and in these are embodied the forces which increase moral and scientific power.

